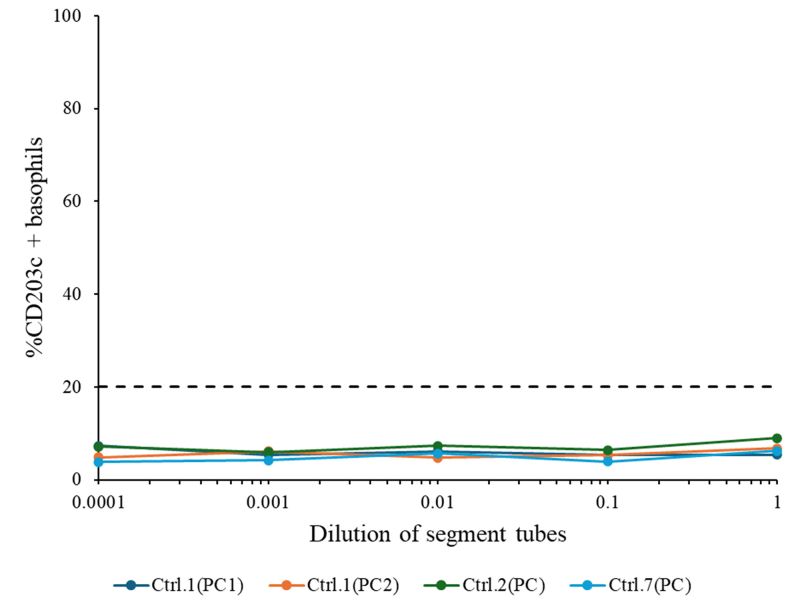# Prospective study of basophil activation test in suspected perioperative transfusion anaphylaxis

**DOI:** 10.1016/j.jacig.2025.100634

**Published:** 2025-12-20

**Authors:** Yasuhiro Amano, Tasuku Fujii, Natsumi Kameyama, Takahiro Tamura

**Affiliations:** a Department of Anesthesiology, Nagoya University Graduate School of Medicine, Nagoya, Japan; b Department of Clinical Laboratory, Nagoya University Hospital, Nagoya, Japan

**Keywords:** Anaphylaxis, basophil activation test, blood products, general anesthesia, transfusion reaction

## Abstract

**Background:**

Identifying the causative blood product in cases of suspected perioperative transfusion anaphylaxis is challenging because no confirmatory skin tests exist, and few blood-based diagnostic tests are available, potentially resulting in underreporting. Although recent studies have demonstrated the utility of the basophil activation test (BAT) for examining the causative relationship between allergic reactions and blood product transfusion, its applicability in suspected perioperative transfusion anaphylaxis remains unknown.

**Objective:**

We aimed to assess the utility of the BAT for suspected perioperative transfusion anaphylaxis cases and calculated the incidence of perioperative transfusion anaphylaxis.

**Methods:**

We prospectively performed BATs using blood products from patients with suspected transfusion anaphylaxis undergoing general anesthesia at a single hospital over 4 years. Transfusion anaphylaxis was confirmed on the basis of a positive BAT and hypersensitivity score.

**Results:**

The hypersensitivity clinical scores in all 11 patients were >8 points. BATs of blood products revealed 6 patients (55%) with positive results and dose–response curves with increasing concentrations of one blood product. These findings confirmed the diagnosis of transfusion anaphylaxis. The overall incidence of perioperative anaphylaxis due to blood products was 1/2,230, 19-fold higher than that in the 6th National Audit Project survey. The highest incidence was associated with fresh frozen plasma (1/670; 95% confidence interval, 1/2,061-1/287), followed by platelet concentrate (1/1,292; 95% confidence interval, 1/51,031-1/232).

**Conclusions:**

The BAT may help identify causative blood products in suspected perioperative transfusion anaphylaxis cases. Moreover, perioperative anaphylaxis may occur more frequently than previously reported.

## Graphical abstract

**Figure:**
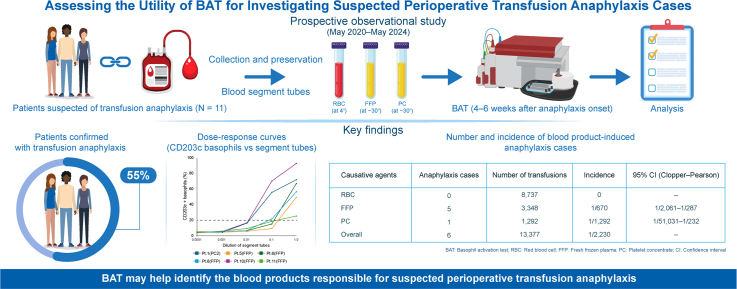

Although blood products are essential for treating anemia or increasing coagulation factor and platelet levels, they can trigger various adverse reactions.^1^ Allergic reactions are the most common transfusion reactions; although rare, severe allergic reactions or anaphylaxis can be life-threatening.^2^ Transfusion anaphylaxis is suspected if symptoms suggestive of anaphylaxis occur during or shortly after transfusion.^3^ Administering medications during transfusion or simultaneously administering blood components is not recommended unless critical bleeding occurs because it is difficult to ascertain whether the medication or the blood component is responsible for the adverse effect.^4^ In routine clinical practice, when anaphylaxis occurs during or shortly after transfusion, the causative blood product can often be identified on the basis of the time interval between transfusion and symptom onset, even without allergy testing.

In contrast, during the perioperative period, multiple drugs are administered, and numerous differential diagnoses can complicate the diagnosis of perioperative anaphylaxis.^5^ Although perioperative anaphylaxis may be suspected on the basis of the onset delay between possible offending agents and clinical signs, this timing should not be solely used to identify the culprit.^6^ Thus, allergologic assessment is essential for identifying the causative agent and preventing the recurrence of anaphylaxis.^7^ However, with current diagnostic tests, determining whether blood products cause perioperative anaphylaxis is challenging. No confirmatory skin tests for blood products have been described.^8^ Although the IgA levels of patients experiencing anaphylactic reactions to blood products should be measured,^9^ findings of IgA deficiency do not support a causal relationship between anaphylaxis and transfusion.^10^^,^^11^ Recently, the basophil activation test (BAT) has been applied to blood products to assess the causative relationship between reactions and transfusion.^12^, ^13^, ^14^ However, in the perioperative period, only two case reports have described the use of the BAT for diagnosing anaphylaxis caused by methylene blue–treated fresh frozen plasma (FFP).^15^^,^^16^ Thus, the usefulness of the BAT for identifying causative blood products in suspected cases of perioperative anaphylaxis remains unclear. Furthermore, we previously reported that perioperative transfusion anaphylaxis may be underreported^17^ and that its incidence can be correctly assessed if the causative blood products are identified.

This study aimed to assess the utility of the BAT in identifying causative blood products in cases of suspected perioperative transfusion anaphylaxis. To ensure the quality of the confirmatory diagnosis of transfusion anaphylaxis, we combined positive BAT results for blood products with the recently developed Hypersensitivity Clinical Scoring Scheme (HCSS).^18^ We also calculated the incidence of perioperative anaphylaxis caused by blood products, which we based on confirmed cases of transfusion anaphylaxis.

## Methods

### Patient selection

This prospective observational study was conducted at Nagoya University Hospital after obtaining approval from the local ethics committee (approval 2020-0020, approved May 13, 2020). Written informed consent was obtained from all participants before study inclusion. This study included all patients suspected of having anaphylaxis caused by blood products (red blood cells [RBCs], FFP, and platelet concentrate [PC]) during general anesthesia between May 2020 and May 2024. Patients without segment tubes containing suspected causative blood products and those with unstable postoperative conditions were excluded.

### Measurement of tryptase and preservation of segment tubes

We obtained patient blood samples within 2 hours and 24 hours after onset of anaphylaxis to measure peak and baseline serum tryptase levels. Values greater than [(1.2 × baseline tryptase) + 2] μg L^−1^ were defined as significant elevations.^19^ Each segment tube of the suspected causative blood products was preserved within 24 hours after onset of anaphylaxis as follows: (1) RBCs stored at 4°C; (2) plasma stored at −30°C; and (3) PC centrifuged and supernatants stored at −30°C.

### BAT

Whole blood was obtained from each patient at least 4 to 6 weeks after onset of anaphylaxis. The blood samples were collected in heparinized tubes, and the BAT was performed using a Navios EX flow cytometer (Beckman Coulter, Brea, Calif) as detailed elsewhere.^20^ An Allergenicity kit (Beckman Coulter) was used within 4 hours of blood sampling. For each patient, the BAT used segment tubes—containing the suspected causative blood products—from that patient’s transfusion. FFP and PC segment tubes were thawed immediately before the BAT. The segment tubes were serially diluted with normal saline (1, 1:10, 1:100, 1:1,000, and 1:10,000); 20 μL of negative control (phosphate-buffered saline [PBS]), positive control (anti-IgE antibody), and blood products were then added to 100 μL blood sample aliquots. The samples were then stained with 20 μL of a mixture containing anti–CRTH2 (chemoattractant receptor-homologous molecule expressed on T_H_2 cells)-fluorescein isothiocyanate (aka FITC), anti–CD203-phycoerythrin, and anti–CD3–phycoerythrin–cyanine 7 (aka PC7) conjugated antibodies. After incubation at 37°C for 15 minutes, 100 μL of stop solution was added. RBCs were lysed, and white blood cells were fixed for 10 minutes at room temperature. After centrifugation, 3 mL of wash solution (PBS) was added to the cell pellets. After the second centrifugation, the cell pellets were resuspended in 0.5 mL of PBS with 0.1% formaldehyde. Basophils were selected on the basis of a side scatter and low CD3^−^/CRTH2^+^ gate profile, and at least 500 basophils were counted. To calculate the percentage of CD203c-positive basophils in the whole blood sample in each panel, a cursor was placed on the CD203c histogram at the point at which 5% of the basophils were CD203c positive without stimulation. Samples with a net percentage of activated basophils >20% were considered BAT positive.^21^

### BAT in healthy volunteers

Because the suspected causative blood product from the patient can activate the basophils of healthy volunteers,^22^ we also enrolled one healthy volunteer for each enrolled patient as a control. The control volunteer had the same blood type as the patient, had no history of blood transfusion, and underwent the BAT using the same segment tubes as we just described.

### Confirmed cases of transfusion anaphylaxis

We defined confirmed cases of transfusion anaphylaxis as those meeting the following two criteria: first, positive BAT result for a segment tube of suspected causative blood products, and second, a HCSS score of >8 points.^18^ All confirmed cases of transfusion anaphylaxis were reported to the Japanese blood bank, and IgA or haptoglobin deficiency was investigated using the patient’s residual pretransfusion serum samples. We also investigated the history of transfusion and reaction, as well as whether other blood products were transfused again and whether allergic transfusion reactions reoccurred.

### Statistical analysis

We collected information regarding the total number of transfusions administered during the study period. The overall incidence of perioperative anaphylaxis due to blood products was calculated as the number of confirmed cases of transfusion-induced anaphylaxis divided by the total number of transfusions. The incidence of anaphylaxis caused by each blood product was calculated on the basis of the cases of confirmed transfusion anaphylaxis. The 95% confidence interval (CI) for incidence was calculated by the Clopper-Pearson method in R v4.4.1 software (R Foundation for Statistical Computing, www.r-project.org).

## Results

**Fig 1:**
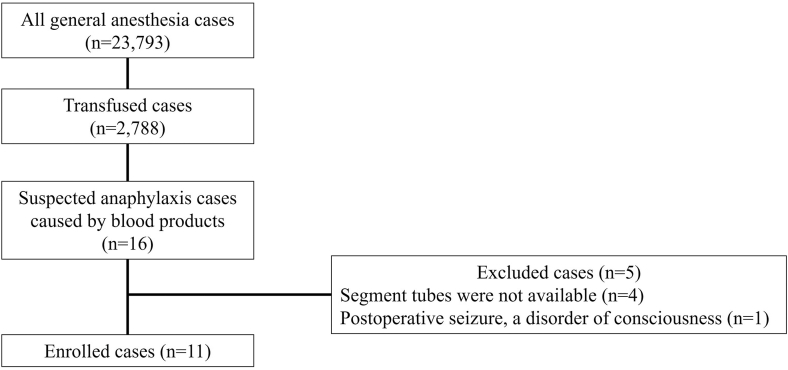
Inclusion and exclusion criteria for suspected cases of perioperative transfusion anaphylaxis.

**Supplementary Fig E1:**
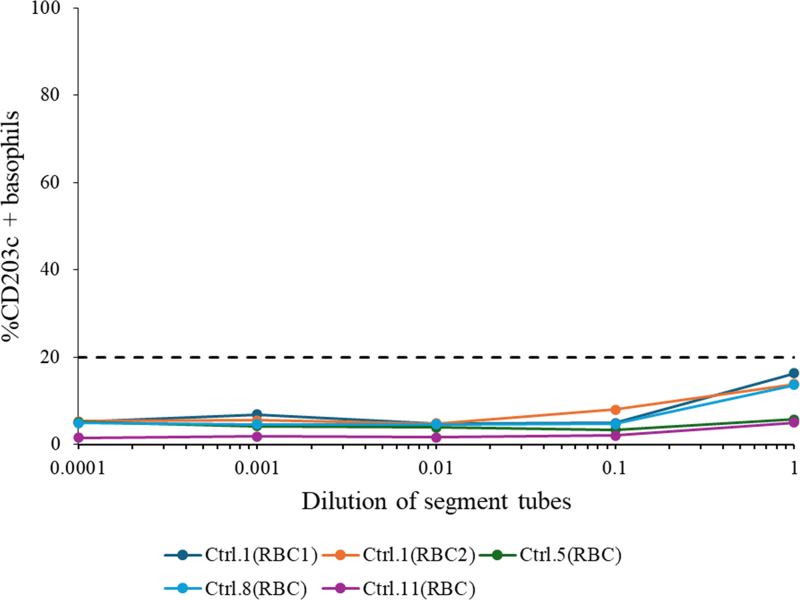


**Supplementary Fig E2:**
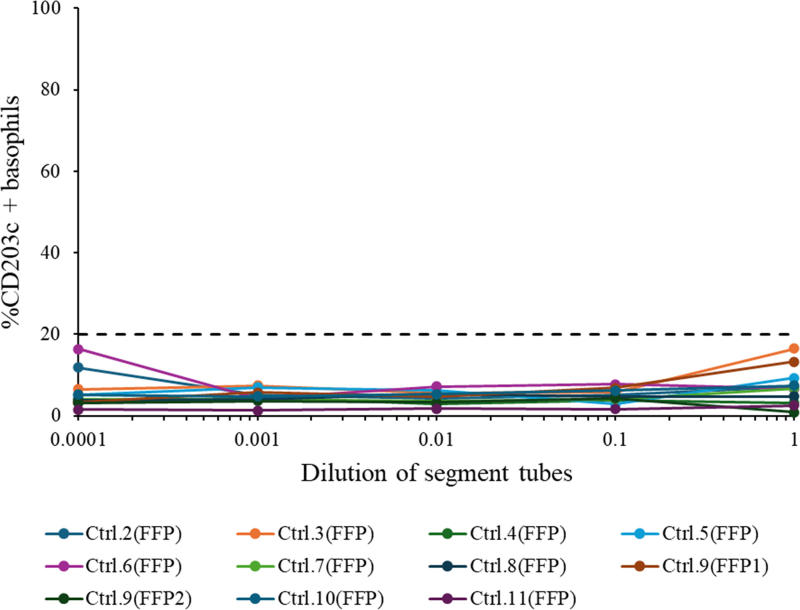


Among the 23,793 patients undergoing general anesthesia, 2,788 received a transfusion, with 16 patients suspected by anesthesiologists of experiencing blood product–induced anaphylaxis. Four patients were excluded because of the unavailability of segment tubes; one was excluded because of prolonged postoperative disturbance in consciousness and seizures. After excluding these 5 patients, the present study included 11 cases of suspected transfusion anaphylaxis (Fig 1). The HCSS scores in all 11 patients were >8 points, and 6 patients showed significantly elevated serum tryptase levels. One to 4 packs of blood products were suspected as the causative agents for each patient, with a total of 20 packs identified. The most commonly suspected product was FFP (11 packs in 10 patients), followed by RBCs (5 packs in 4 patients) and PC (4 packs in 3 patients). For each patient, 1 to 4 segment tubes were used for the BAT, with a total of 20 segment tubes used in 11 patients and their respective controls. Of the 20 segment tubes, 6 (1 PC and 5 FFP) showed CD203c basophil activation >20% among 6 patients; these findings confirmed the diagnosis of blood product–induced anaphylaxis. The remaining 5 patients were diagnosed with uncertain transfusion anaphylaxis (Table I). All 20 segment tubes did not show activation of >20% in the 11 controls (see Figs E1-E3 in this article’s Online Repository available at www.jaci-global.org).

**Table I:** Test results for patients with suspected transfusion anaphylaxis

Patient no.	Age (years)	Sex	Tryptase (μg L−^1^)		HCSS score	Suspected causative blood product	Activated CD203c basophils in positive control (%)	Activated CD203c basophils in segment tubes of suspected causative agents (%)	Diagnosis	Time to onset after administration of causative blood product (minutes)
			Peak	Baseline						
1	50	F	7.8	6.3	12	PC1, PC2, RBC1, RBC2	91.1	5.5 (PC1), 72.1 (PC2), 11.5 (RBC1), 11.0 (RBC2)	Anaphylaxis caused by PC	24
2	75	M	2.6	2.4	9	FFP, PC	14.1	8.1 (FFP), 6.3 (PC)	Transfusion anaphylaxis uncertain	—
3	81	M	26.2	8	28	FFP	74.7	9.3 (FFP)	Transfusion anaphylaxis uncertain	—
4	54	M	4.1	2.2	13	FFP	47.1	6.9 (FFP)	Transfusion anaphylaxis uncertain	—
5	73	M	19.9	6.1	29	FFP, RBC	94.9	49.0 (FFP), 9.0 (RBC)	Anaphylaxis caused by FFP	18
6	76	F	4.5	3.1	15	FFP	82.5	67.0 (FFP)	Anaphylaxis caused by FFP	9
7	61	M	7	3.1	15	FFP, PC	67.7	8.8 (FFP), 4.4 (PC)	Transfusion anaphylaxis uncertain	—
8	67	M	10.9	4.6	18	FFP, RBC	90.3	57.1 (FFP), 10.3 (RBC)	Anaphylaxis caused by FFP	17
9	73	M	10.3	4.2	8	FFP1, FFP2	84.9	4.4 (FFP1), 4.4 (FFP2)	Transfusion anaphylaxis uncertain	—
10	80	M	4.9	3.6	15	FFP	98.7	92.9 (FFP)	Anaphylaxis caused by FFP	9
11	50	M	15.3	5.4	31	FFP, RBC	97.5	25.7 (FFP), 14.5 (RBC)	Anaphylaxis caused by FFP	9

Consecutive numbers were assigned if more than one causative blood product was suspected. Shown is proportion of activated basophils when basophils were most highly activated.

### Patients with confirmed transfusion anaphylaxis

**Fig 2:**
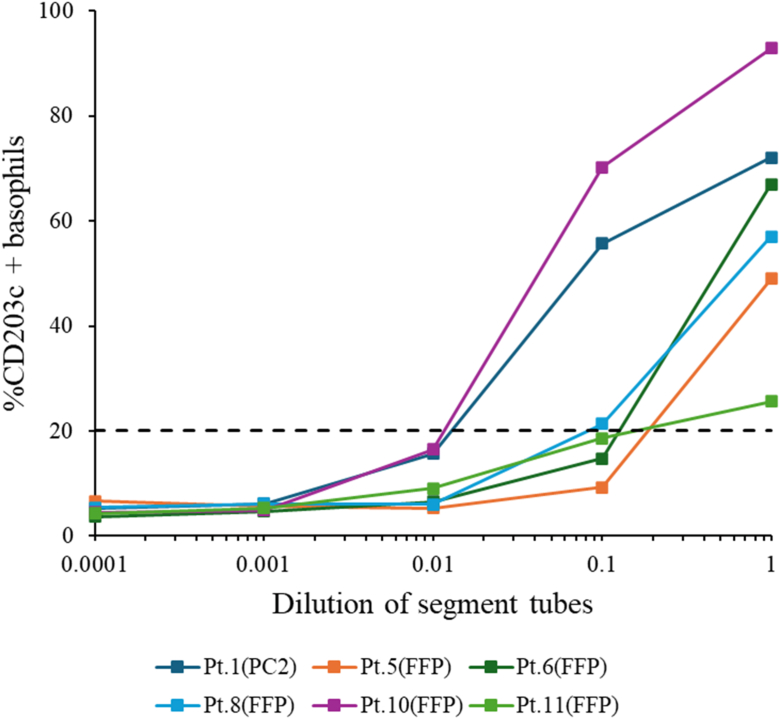
Results of BATs with CD203c of 6 patients with confirmed transfusion anaphylaxis. *Dashed line* denotes positivity threshold (20%). All 6 patients showed positive results and dose–response curves of basophil activation with increasing dilutions of each suspected segment tube. *Pt,* Patient.

In the 6 patients with confirmed transfusion anaphylaxis, CD203c basophil activation showed a dose–response curve with increasing segment tube concentrations (Fig 2). CD203c basophils in 3 segment tubes were >20% with a 1:10 dilution, and all CD203c basophils in 6 undiluted segment tubes were >20%.

Of the 6 patients, 3 had a history of transfusion with no reaction. All 6 patients with confirmed transfusion anaphylaxis underwent transfusion again 1 to 7 days after onset of anaphylaxis, 4 of whom were retransfused with the same type of culprit blood product. None of the patients experienced allergy recurrence. No IgA or haptoglobin deficiency was detected (Table II).

**Table II:** Transfusion and reaction history of patients with confirmed transfusion anaphylaxis

Patient no.	History of transfusion and reaction	Diagnosis	IgA deficiency	Hp deficiency	Retransfusion of blood products	Allergy recurrence after retransfusion
1	Yes, no reaction	Anaphylaxis caused by PC	No	No	RBC, PC	No
5	Never	Anaphylaxis caused by FFP	No	No	RBC, FFP	No
6	Never	Anaphylaxis caused by FFP	No	No	RBC	No
8	Never	Anaphylaxis caused by FFP	No	No	RBC, PC	No
10	Yes, no reaction	Anaphylaxis caused by FFP	NA	NA	RBC, FFP	No
11	Yes, no reaction	Anaphylaxis caused by FFP	No	No	RBC, FFP, PC	No

Pretransfusion serum from confirmed transfusion cases was used to measure IgA levels or haptoglobin deficiency.

*Hp,* Haptoglobin; *NA,* not available.

### Incidence of blood product–induced perioperative anaphylaxis

The overall incidence of blood product–induced perioperative anaphylaxis was 1/2,230 (Table III). The highest incidence was associated with FFP (1/670; 95% CI, 1/2,061-1/287), followed by PC (1/1,292; 95% CI, 1/51,031-1/232). RBCs were the most commonly transfused blood products, but no cases of confirmed anaphylaxis caused by RBCs were observed.

**Table III:** Number and incidence of blood product–induced cases of anaphylaxis

Causative agent	No. of anaphylaxis cases	No. of transfusions	Incidence	Clopper-Pearson 95% CI
RBC	0	8,737	0	—
FFP	5	3,348	1/670	1/2,061 to 1/287
PC	1	1,292	1/1,292	1/51,031 to 1/232
Overall	6	13,377	1/2,230	—

## Discussion

Among the 11 patients with suspected transfusion anaphylaxis, 6 (55%) were diagnosed with anaphylaxis caused by blood products on the basis of positive BAT results. Because no commercial skin tests exists that can confirm blood products,^8^ specific IgE cannot be measured; moreover, the provocation test is difficult. We applied the HCSS to objectively assess the certainty of the clinical diagnosis of transfusion anaphylaxis, which exceeded 8 points in all 11 patients. However, an HCSS score exceeding 8 points did not confirm that the case was truly transfusion anaphylaxis. Among the 11 patients, anaphylaxis could have been caused by causative agents other than blood products in some cases. Thus, if the BAT is performed in true transfusion anaphylaxis cases, the BAT positivity rate could be 55% or higher. To our knowledge, only two case reports have described perioperative transfusion anaphylaxis, which was caused by methylene blue–treated FFP.^15^^,^^16^ Because more than half the patients in our study showed positive BAT results, the BAT may be useful for identifying the causative blood products in cases of suspected perioperative transfusion anaphylaxis.

Although our findings suggest that the BAT could help identify the causative transfused blood products, several issues need to be addressed before using the BAT to determine whether further transfusion is safe during the perioperative period. As recommended, we performed the BAT at least 4 to 6 weeks after anaphylaxis.^23^ However, another blood product was retransfused safely in 6 patients with confirmed transfusion anaphylaxis within 1 to 7 days after onset of anaphylaxis. Thus, retransfusion was performed before obtaining the BAT results. In the perioperative period, further transfusion may be urgently needed, and withholding all transfusions poses a heightened risk of surgical bleeding or coagulopathy. Therefore, standard components should be transfused under direct monitoring in a clinical area with resuscitation facilities.^9^ We believe that emergency blood transfusions should not be withheld until the BAT is completed. Akiki et al in 2023 reported the good negative predictive value of the combination of the BAT and skin tests to determine the safety of subsequent transfusion.^24^ When the next transfusion is not urgently required, this combination may be useful in the perioperative period to predict whether the next product can be transfused safely.

Unlike other drugs, because blood products are unique and have no substitutes, obtaining residual suspected causative agents before discarding blood samples is crucial. Moreover, because the BAT requires fresh drugs,^25^ residuals of suspected blood products must be stored properly until testing. The shelf life of PC is 3 days, and we preserved the supernatants of PC as described previously.^12^^,^^21^ Because we wished to perform the BAT for RBCs to mimic clinical practice, they were preserved using the same storage methods as before transfusion in the clinical setting. All segment tubes of RBCs had expired when the BAT was performed, which may have affected basophil activation. However, all 4 cases in which RBCs were included as suspected causative products yielded positive BAT results for PC or FFP (Table I). The residual suspected causative blood product consisted of a blood bag and a segment tube. Because bacteria could enter the blood bag through the blood administration port and affect the BAT results, we used segment tubes. Moreover, because the test requires only a small amount of tested drug, segment tubes are sufficient to perform the BAT. We also enrolled controls with the same blood type as the patients to avoid unnecessary hemolytic reactions that may also have affected the BAT results.

The suspected factors for transfusion anaphylaxis can be classified as donor and patient factors. The potential donor factors include biologic response modifiers released from platelets and damage-associated molecular patterns (known as DAMPs) such as mitochondrial DNA, antibodies against IgE, or IgE aggregates present in donor plasma.^26^ Because the 6 segment tubes that activated basophils in the 6 patients did not activate basophils in the 6 healthy volunteers, it is unlikely that donor factors alone were the causative agents of transfusion anaphylaxis. The potential patient factors include allergic predisposition, primed basophils and/or mast cells, hematologic and malignant diseases, DAMPs, IgA or haptoglobin deficiency and antibodies to them, methylene blue or food allergen, or an unidentified allergen.^26^ Among the 6 patients with confirmed transfusion anaphylaxis, 1 had hay fever and 3 underwent surgery for malignant tumors. None of the patients had IgA or haptoglobin deficiencies (Table II). However, methylene blue–treated blood products are not commercially available in Japan. Because this study did not examine the immunologic pathway of transfusion anaphylaxis, whether the 6 confirmed cases of transfusion anaphylaxis were caused by donor or patient factors was unclear. Because all 6 patients were BAT positive for only one specific product and all 6 were safely retransfused, anaphylaxis may have occurred for certain donor/recipient combinations.

The results of this study also showed an overall incidence of transfusion anaphylaxis of 1/2,230, with the highest incidence associated with FFP (1/670), followed by PC (1/1,292). The causative agents and the incidence of perioperative anaphylaxis caused by the culprit agents vary among countries.^8^ Neuromuscular blocking agents and antibiotics are the most common causative agents worldwide, and sugammadex is common in Japan.^27^ The incidence of perioperative anaphylaxis caused by neuromuscular blocking agents, antibiotics, and sugammadex in recent nationwide studies is approximately 1/9,000 to 1/27,000.^27^, ^28^, ^29^ Several epidemiologic studies of perioperative anaphylaxis have included several cases of transfusion anaphylaxis.^29^, ^30^, ^31^ Only the 6th National Audit Project estimated the incidence of anaphylaxis caused by FFP and cryoprecipitate (1/42,000).^29^ Surprisingly, the incidence of perioperative anaphylaxis caused by FFP and the overall incidence of perioperative anaphylaxis caused by blood products in the present study were 63- and 19-fold higher, respectively, than those in the 6th National Audit Project survey. The reason for the high incidence rates in the present study remains unknown. However, the incidence of blood products in our study was unlikely to be overestimated because we only calculated the incidence with a definitive diagnosis of transfusion anaphylaxis combined with positive BAT results and the HCSS. However, the incidence may be even higher because of the risk of false-negative BAT results. Moreover, during the perioperative period, multiple blood products are often transfused to a single patient, so anaphylaxis is even more likely to occur.

This study has several limitations. First, in the 5 patients with negative BAT results, falsely negative results, or presence of causative agents other than blood products could not entirely be excluded. Ideally, all drugs administered before the onset of anaphylaxis should be investigated. However, in the perioperative setting, transfusion is typically performed several hours after anesthesia induction. When signs of anaphylaxis appear after transfusion, it is thus reasonable, just on the basis of timing, to suspect the blood product. In addition, testing all agents administered during the perioperative period would impose a considerable burden on the patient. Moreover, a negative result for all agents other than the blood product does not establish causality; definitive attribution requires a positive result specifically linked with the blood product in question. For these reasons, we focused on blood products and did not routinely investigate other perioperatively administered drugs. We consider it reasonable to initially perform the BAT with blood products when transfusion-related anaphylaxis is suspected, and to subsequently examine other drugs with skin tests or alternative methods if the BAT result is negative. Second, the sensitivity and specificity of the BAT for transfusion-related anaphylaxis remain to be determined, and including only true transfusion cases to assess them is often challenging. Finally, this was a single-institution study with a small number of patients; therefore, future multicenter prospective studies are warranted.

In conclusion, the results of this study demonstrated that the BAT could contribute to the identification of the causative blood products in cases of suspected perioperative transfusion anaphylaxis. Moreover, perioperative anaphylaxis occurred more frequently than previously reported. Further studies are needed to better investigate cases of suspected perioperative transfusion anaphylaxis.

**Key messages**

• The BAT is useful for identifying the causative blood product in cases of suspected transfusion anaphylaxis.
• Perioperative transfusion anaphylaxis occurs more frequently than reported.

## Disclosure statement

Disclosure of potential conflict of interest: The authors declare that they have no relevant conflicts of interest.

## Figures and Tables

**Supplementary Fig E3:**