# Supplementary material for: Prospective study of basophil activation test in suspected perioperative transfusion anaphylaxis
Source: J Allergy Clin Immunol Glob. 2025 Dec 20;5(2):100634. doi: 10.1016/j.jacig.2025.100634 (PMC12830261; doi:10.1016/j.jacig.2025.100634)
Supplement: Supplementary Data [file mmc1.docx]

**Supplementary Figure Legend**

**Supplemental Figure 1.** Results of basophil activation tests with CD203c of four controls with five segment tubes of red blood cells. The dashed line denotes the positivity threshold (20%). Ctrl, Control.

**Supplemental Figure 2.** Results of basophil activation tests with CD203c of ten controls with 11 segment tubes of fresh frozen plasma. The dashed line denotes the positivity threshold (20%). Ctrl, Control.

**Supplemental Figure 3.** Results of basophil activation tests with CD203c of three controls with four segment tubes of platelet concentrate. The dashed line denotes the positivity threshold (20%). Ctrl, Control.
